# Impact of sowing date and level of phosphorus application on economic returns in cotton

**DOI:** 10.3389/fpls.2024.1402731

**Published:** 2024-06-12

**Authors:** Jacob Shauri Tlatlaa, George Muhamba Tryphone, Eliakira Kisetu Nassary

**Affiliations:** ^1^ Sokoine University of Agriculture, College of Agriculture, Department of Crop Science and Horticulture, Chuo-Kikuu, Morogoro, Tanzania; ^2^ Sokoine University of Agriculture, College of Agriculture, Department of Soil and Geological Sciences, Chuo-Kikuu, Morogoro, Tanzania

**Keywords:** agricultural sustainability, cotton productivity, economic viability, environmental sustainability, nutrient management, rural agriculture

## Abstract

This study explored the economic dynamics of cotton (*Gossypium hirsutum* L.) production in Msilale village, Chato District, Tanzania. The experiment utilized a factorial design with sowing dates on November 25^th^, December 15^th^, and January 4^th^, and phosphorus levels at 0, 20, 40, and 60 kg P ha^-1^, replicated three times. Results indicated significantly higher cotton yields (6.1 t ha^-1^ and 6.3 t ha^-1^) for November and December sowings compared to January (3.8 t ha^-1^). This is a 61% and 66% increase in cotton yields for November and December sowings, respectively relative to January sowing. Though not significant, 20 kg P ha^-1^ and 40 kg P ha^-1^ applications yielded 5.8 t ha^-1^ and 5.4 t ha^-1^, respectively, while 60 kg P ha^-1^ yielded 5.3 t ha^-1^. This is a 9.4% and 1.9% increase in cotton yields at 20 and 40 kg P ha^-1^, respectively relative to absolute control and 60 kg P ha^-1^ application. Economic analysis revealed that late sowing (January) had the lowest net profit (Tshs. 3,723,400 ≈ USD 1,486) and benefit-to-cost ratio (BCR) of 11.2. Early sowings recorded higher net profits (Tshs. 6,679,527 ≈ USD 2,666 and Tshs. 6,861,283 ≈ USD 2,738) and BCRs (18.4 and 18.8, respectively). This is a 79% (BCR = 64%) and 84% (BCR = 68) increase in net benefits from early sowings compared to late sowing. Applications of 20, 40, and 60 kg P ha^-1^ resulted in net benefits of Tshs. 5,452,572 ≈ USD 2,176 (BCR = 19.2), Tshs. 5,209,904 ≈ USD 2,079 (BCR = 15.1), and Tshs. 5,748,786 ≈ USD 2,294 (BCR = 14.1), respectively, with a significant (p = 0.017) BCR at 20 kg P ha^-1^ indicating cost-effectiveness. This is a 36% and 7.1% economic benefit at 20 and 40 kg P ha^-1^, respectively compared to 60 kg P ha^-1^ application. Optimizing sowing dates and P levels can boost economic returns in cotton production and promote sustainability.

## 1 Introduction

Cotton (*Gossypium hirsutum* L.) production is a critical component of global agriculture, playing a pivotal role in textile and apparel industries, as well as providing a source of livelihood for millions of small-scale farmers around the world (Kedisso et al., 2023; Voora et al., 2023). However, the economic benefits derived from cotton production can vary significantly depending on various factors, including sowing dates and the application of essential nutrients like nitrogen (N) and phosphorus (P) (Hussain et al., 2022; Shah et al., 2022; van der Sluijs, 2022; Li et al., 2023). The importance of cotton industry cannot be underestimated in the global economic. Cotton accounts for ~ 7% of the agricultural value-addition, high contribution to foreign exchange (60%), edible oil (64%) and 1.4% of the GDP, and employing about 40% of the labour-force (Liu et al., 2013; Ullah et al., 2019). In Tanzania, cotton by-products find diverse applications: cotton stalks are utilized in pellet and briquette production for heating, while cottonseedcake serves as an organic soil amendment (Kabissa, 2016). Additionally, cotton cultivation in Tanzania significantly contributes to export earnings and provides employment opportunities for rural households, supporting the country’s socio-economic development (Altenbuchner et al., 2016).

In Tanzania, total cotton productions per season are about 150,000 tons of seeds and 78,000 tons of lint from land area ranging from 350,000 to 550,000 hectares (FAOSTAT, 2018; TCB, 2018). Cotton production in Tanzania is more concentrated in the Western Cotton Growing Area (WCGA) contributing about 95% of the total production and the Eastern Cotton Growing Area (ECGA) accounts for 5% of the total production. The regions in WCGA include Mwanza, Shinyanga, Simiyu, Geita, Mara, Kigoma, Tabora, Kagera, Singida, Katavi, and parts of Dodoma region, while the ECGA covers Morogoro, Kilimanjaro, Manyara, Tanga, Coast and parts of Iringa regions (TCB, 2018). Reports show that cotton yields in Tanzania consistently fall well below their potential of 1.5 t ha^-1^, typically ranging from 6 to 8 t ha^-1^, largely due to irregular sowing dates (TCB, 2018). In Chato district of Geita region, the prevalent cultivation of the UKM-08 cotton variety, bred for high yields of around 4 t ha^-1^, results in an average yield of 0.3 t ha^-1^, indicating a significant loss of over 3.7 t ha^-1^ of seed cotton yield (Kabissa, 2016). Soil nutrient deficiencies, notably in N and P, negatively impact amino acid and protein synthesis, thereby reducing fibre quality. Phosphorus deficiency in soils also correlates with decreased biomass accumulation, leading to lower seed cotton yields. The application of phosphatic fertilizers is widely recognized to enhance both cotton yield and fibre quality (Romero-Perdomo et al., 2021). The documented blanket recommendations for N, P, and potassium (K) nutrients for cotton production in Tanzania, Kenya, and Uganda range from 20 to 60 kg N ha^-1^, 15 to 30 kg P ha^-1^, and 30 to 40 kg K ha^-1^, as outlined by Bekunda et al. (1997). However, there is limited documentation regarding fertilizer usage in Tanzanian cotton-growing soils (Msigwa, 2019). Hence, it is essential to evaluate the effects of P application on seed cotton yield and fibre quality, taking into account optimal sowing dates.

To enhance economic benefits for farmers engaged in cotton production on small landholdings, it is imperative to consider a range of strategies that revolve around sowing dates and P application. These strategies need to aim at optimizing yields, minimizing production costs, and ultimately increasing profitability (Mauget et al., 2019; Singh et al., 2020). The choice of sowing date significantly impacts cotton yield and quality (Mudassir et al., 2022). Early sowing leads to higher yields and better fibre quality in cotton, but it can also increase the risk of pest and disease infestations (Singh et al., 2017). Conversely, late sowing may reduce these risks but could result in lower yields (Fei et al., 2022). Climate data and local knowledge is important in identifying the most suitable sowing window that balances trade-offs realized from cotton production (Sankaranarayanan et al., 2020). As climate patterns continue to change, there is a need to investigate how altering sowing dates can help cotton crops adapt to shifting weather conditions (Ahmed et al., 2019; Tun Oo et al., 2023; Wu et al., 2023). Sowing dates are reported elsewhere to translate into economic variations of cotton following differential influence on the number and weight of bolls per individual cotton plant, cottonseed yield, staple length, micronaire, and lint strength (Ullah et al., 2019; Godfrey et al., 2023). Such information is scant under Tanzanian cotton-producing systems (Altenbuchner et al., 2016). Therefore, research is needed that can focus on finding optimal sowing dates that align with changing temperature and precipitation patterns to optimize cotton production and the overriding economic return.

Phosphorus is a crucial nutrient for cotton plants, playing a fundamental role in various physiological processes such as energy transfer, photosynthesis, and nucleic acid synthesis (Luo et al., 2020). It is essential for the development of robust root systems, early crop establishment, and overall plant growth. However, P is often found in limited availability in many agricultural soils, and deficiencies can lead to reduced cotton yields and inferior fibre quality (van de Wiel et al., 2016; Nachimuthu et al., 2022). Conducting soil tests to determine P levels and tailor fertilizer application accordingly is important for higher cotton productivity. In response to the challenge of P deficiency in cotton-growing regions, the use of P-containing fertilizers has become a standard agricultural practice (Luo et al., 2020; Nachimuthu et al., 2022). Phosphorus fertilization is employed to address nutrient imbalances, improve plant vigour, and maximize cotton yield potential (Romero-Perdomo et al., 2021). The effectiveness of P fertilization in cotton production depends on several factors, including the timing and method of application, soil characteristics, and local climate conditions (Weeks and Hettiarachchi, 2019). As cotton cultivation continues to expand and adapt to changing environmental and economic conditions, a deeper understanding of P fertilization’s role becomes increasingly crucial (Gomez-Zavaglia et al., 2020; Malhi et al., 2020; Giller et al., 2021; Dobermann et al., 2022). While this knowledge is lacking for the smallholder farmers in Tanzania, it can guide cotton growers, agronomists, and researchers in optimizing P management practices to achieve sustainable and economically viable cotton production systems (Riar et al., 2019; Constantine et al., 2023). The rationale of the present study was to investigate the economic aspects of cotton production, with a focus on sowing dates and P application. It aimed to explore how varying sowing dates and P application levels and their interactions influenced cotton yield and economic profitability. The objective was to identify optimal combinations of these factors that resulted in maximum yield and economic returns for local cotton farmers. Additionally, the study incorporated an economic analysis component to evaluate the financial implications of different management practices, with inclusion of net profits and benefit-to-cost ratios associated with each treatment combination. Overall, the study aimed to contribute to the development of evidence-based recommendations for improving cotton production practices in Msilale village and Tanzania at large, thereby supporting the economic sustainability of local farming communities. Therefore, the primary hypothesis of the present study was that implementing strategic P application in Tanzanian agriculture in conjunction with optimizing sowing dates could significantly improve cotton yields, fibre quality and economic return.

## 2 Materials and methods

### 2.1 Description of experimental site

Explorations unfolded in the charming village of Msilale, situated within the scenic landscape of Chato District, Tanzania. Positioned between latitudes 02^0^ 15’ and 03^0^ 15’ S, and longitudes 31^0^ and 32^0^ E, this locale exudes a geographical allure. Its elevation, ranging from 1135 meters to 1141 meters above mean sea level, adds a dimension to its captivating profile (Tlatlaa et al., 2023). Msilale experiences the rhythmic dance of rainfall in a bimodal pattern, with short showers gracing the land from September to December, while the long rainy season stretches its benevolent embrace from February to May. The orchestration of nature manifests in an annual rainfall average of 850 mm, creating a harmonious symphony with temperatures oscillating between a delightful 24°C to 30°C (Msigwa, 2019).

Antecedent to this experiment, we collected composite soil samples from the depth of 0–30 cm. These samples, drawn from two distinct field references—Chato-Msilale and Muungano-Kahumo—underwent an exhaustive analysis to unravel the intricate tapestry of various soil properties. A nuanced revelation emerged as the Chato-Msilale soil disclosed a nuanced hint of acidity in its pH, indicating a potential conducive environment for cotton cultivation. Conversely, the Muungano-Kahumo soil showcased elevated levels of organic matter and available P, illuminating prospects for fostering advantageous effects on cotton growth. The intricacies of these findings are encapsulated in the work of Tlatlaa et al. (2023). The discerning decision to anoint Chato-Msilale as the focal point of study sprouted from a meticulous scrutiny of soil conditions, on ameliorating constraints tied to available P levels.

In addition, the selection of Chato District, among many in the country, as the epicentre for this groundbreaking field trial was a judicious confluence of factors. Insightful gleanings from a feasibility study, expounded upon by Tlatlaa et al. (2023), provided a guiding compass. The presence of water for supplementary irrigation, harmonized with the counsel of local agricultural authorities, played pivotal roles in shaping this strategic choice. A rich history of cotton production and the undulating topography were also the crucial factors considered in site selection. This endeavour stands as a pioneering foray in the national arena, as it pioneers the application of fertilizers and manipulation of sowing dates to gauge cotton performance under local idiosyncrasies, with the detailed chronicles of the field experiment documented in Tlatlaa et al. (2023). This initiative sows the seeds for potential future scalability of cotton production, positioning itself as a catalyst for transformative growth in the agricultural landscape.

### 2.2 Experimental design and treatments

The research was structured as a factorial experiment, employing a randomized complete block design (RCBD) that was replicated three times. Two factors, sowing dates and P levels, were examined across different levels. Sowing dates included 25^th^ November 2022 (D1), 15^th^ December 2022 (D2), and 4^th^ January 2023 (D3), while P levels were the control (P0), 20 kg P ha^-1^ (P1), 40 kg P ha^-1^ (P2), and 60 kg P ha^-1^ (P3). The treatments, representing the combination of sowing dates and P levels, were replicated three times for each sowing date, resulting in a total of 36 plots. Farmers in Western Tanzania commonly choose sowing dates between November 15^th^ and December 15^th^ for cotton cultivation, as reported by TCB (2018). However, this prevalent practice has been associated with reduced yields, compromised fibre quality, and diminished economic returns when compared to the outcomes achieved through timed sowing, as highlighted by Msigwa (2019).

The soil analysis revealed slightly acidic soil with normal electrical conductivity but severe deficiencies in total nitrogen and organic carbon. Moreover, both studies identified low cation exchange capacity, indicating limited nutrient-holding capacity, and medium levels of available P. Phosphorus, in the form of diammonium phosphate (DAP, 46% P_2_O_5_), was applied during seed sowing using a localized application method. Holes were made in the soil, DAP was added, and then covered with a thin layer of soil before seeding. The holes were then covered with another layer of soil. Nitrogen was uniformly top-dressed 21 days after sowing using urea (46% N) at a rate of 60 kg N ha^−1^. This was done through a banding application, where the fertilizer was applied around the plant. This application was based on routine soil characterization indicating very low nitrogen and medium phosphorus levels. Cotton seeds of the UKM-08 variety, well-suited for the region and commonly used by local farmers, were sourced from Tanzania Agricultural Research Institute (TARI)-Ukiruguru.

Planting involved two seeds per hole, with a spacing of 0.3 m between plants within rows and 0.6 m between rows, resulting in a plot size of 6.48 m^2^. Each plot consisted of 5 rows with 10 holes per row, totalling 100 plants per plot (equivalent to 154,321 plants per hectare). Plots within a replicate were spaced 0.5 m apart, and replicates were spaced 1 m apart. In drought conditions with uneven rainfall, frequent irrigation was implemented, along with the use of mulching materials to enhance soil water-holding capacity. The study also involved monitoring environmental conditions through a local weather station established two months before the experiment. Data on rainfall and temperature were collected before, during, and after the experimentation period, covering the months from November 2022 to June 2023 (See Tlatlaa et al., 2023). Harvesting took place in May and June 2023 (Tlatlaa et al., 2023).

### 2.3 Data collection

Cotton growth and yield parameters, including plant height, boll number per plant, boll weight, gin turnout, and lint yield, were evaluated in a previous but parallel study (see Tlatlaa et al., 2023). Following statistical analysis, the findings revealed notable disparities in cotton yields across different sowing periods, with November and December 2022 sowings recorded significantly higher yields of 6.1 t ha^-1^ and 6.3 t ha^-1^, respectively, compared to January 2023 sowing, which recorded a yield of 3.8 t ha^-1^. While not statistically significant, plots treated with 20 kg P ha^-1^ and 40 kg P ha^-1^ applications demonstrated yields of 5.8 t ha^-1^ and 5.4 t ha^-1^, respectively, whereas those treated with 60 kg P ha^-1^ yielded 5.3 t ha^-1^. In contrast, plots without P application yielded within the range of 5.1–5.4 t ha^-1^ (Tlatlaa et al., 2023). Thereafter, the current study conducted a comprehensive analysis of the benefits of incorporating P in cotton production, examining variations within specific sowing dates and across different time frames. The economic impact of phosphorus application was assessed through a partial budget analysis, acknowledging the assumption that the market price of phosphorus-containing fertilizer fluctuated across the various sowing dates.

The assessment of P’s economic influence on cotton centred on the disparity between the incomes derived from selling cotton seeds harvested from plots where P was applied, juxtaposed with yields from plots devoid of P application. To establish a baseline, the incomes from control plots were aggregated into a single mean, serving as a reference for estimating the actual effects of each P level applied to individual replicates. Various P levels were then systematically tested against the average yield of control plots with no P application.

The collected data encompassed the computation of total variable costs (TVC), which encompassed expenses related to seed acquisition, land preparation, labour (for activities such as sowing, weeding, irrigation, and the application of fertilizer, insecticide, and fungicide), as well as harvesting. Additional information included the market value (Tanzanian Shillings 1,060 per 1 kg of cotton, equivalent to USD 0.423) at the farm level during the harvesting period. The actual harvest plot area was standardized at 6.48 m^2^ (with 1 hectare approximately equal to 104 m^2^). All other costs associated with cotton production remained constant across different P application levels within a specific sowing date, with the exception of the absolute control plots. Given that other costs were uniform across experimental plots, the plots which received P-containing fertilizers had additional costs related to fertilizer purchasing and labour for fertilizer application (**Table 1**). It is noteworthy that costs related to the transportation of fertilizer to the field were excluded from the analysis, given that the agro-dealer facilitated direct delivery during the application period. The market value of cotton harvested from each experimental plot was calculated using the formula specified in Equation 1. Information regarding cotton growth, fibre yield, and gin turnout percentage has been detailed in a prior study conducted by Tlatlaa et al. (2023). This earlier research serves as a foundation and background for the current study.

**Table 1:** Input costs of fertilizer purchasing and labour for fertilizer application.

Rate	Bags each 50 kg	^a^Cost/bag	Cost/rate	Mandays/plot	Cost per plot	Total cost/rate	Cost/rate
(kg P/ha)	(bags/ha)	(Tshs)	(Tshs)		(Tshs)	(Tshs)	(USD)
0	0	–	–		–	–	–
20	2	52,000.00	105,144.00	0.0052	38.88	105,182.88	42.01
40	4	52,000.00	210,288.00	0.0052	38.88	210,326.88	84.00
60	6	52,000.00	315,432.00	0.0052	38.88	315,470.88	125.99

**^a^** The cost is based on government’s fertilizer subsides; *Exchange rate 1 USD = Tshs. 2,505.9= on 13^th^ September 2023.


(1) $$Q=\frac{A\times 1,060\times 10^{4}}{6.48}$$

Where Q is the gross benefit (GB) derived from selling of a certain amount of cotton harvested in a specific plot (but extrapolated into hectare basis), and *A* is the amount of cotton harvested in a plot.

The current market value of cotton at the harvest stage was determined, and subsequent to this, the benefit-to-cost ratio (BCR) and marginal return (MR) were computed utilizing the methodology outlined by Shaaban and Kisetu (2014), as expressed in Equation 2. This calculation framework serves as the basis for evaluating the economic aspects of the harvested cotton in the context of the study.


(2) $$\text{Benefit Cost Ratio }(BCR)=\frac{\text{Gross Benefit }(GB)}{\text{Total Variable Cost }(TVC)}$$

Should the benefit-to-cost ratio (BCR) fall below 1, it signifies that the costs associated with the examined P levels outweigh the corresponding benefits. Conversely, if the BCR surpasses 1, it indicates that the benefits derived from the tested P levels outweigh the incurred costs. This threshold analysis provides a valuable insight into the economic viability of the P application under consideration.

The analysis assessing P utilization on cotton and its implication on economic returns was conducted based on the following set of assumptions: (1) Market price variation: The study assumes that the market price of P-containing fertilizer is not constant across different sowing dates. This implies that variations in market prices during different sowing periods might influence the economic outcomes and should be considered in the analysis. (2) Income calculation methodology: The study assumes that the impact of P on the economics of cotton is accurately represented by the difference in incomes generated from selling cotton seed harvested from P-applied plots compared to yields of plots without P application. This assumes that income is a reliable indicator of economic benefits and that the chosen methodology effectively captures the economic impact of P application. (3) Control plot representation: The study assumes that combining the incomes from replicates of control plots to calculate a single mean is a valid approach for estimating the actual effect of each level of P applied to each replicate. This assumes that the control plots are homogenous and that variations within them are negligible for the purpose of analysis. (4) P level testing: The study assumes that varying levels of P were appropriately tested against the average yield of control plots where no P was applied. This assumes that the control plots adequately represent the baseline and provide a valid basis for comparison. (5) Cost consistency: The study assumes that all costs of cotton production, excluding fertilizer transportation, are constant across all levels of P application within a specific sowing date. This implies that factors such as labour, land preparation, and other inputs have consistent costs, which may impact the accuracy of economic assessments. (6) Market value conversion: The study assumes a constant market value conversion of Tshs. 1,060 per 1 kg of cotton, equivalent to USD 0.423. This assumes that the exchange rate and market conditions remain stable during the harvesting period. (7) Exclusion of fertilizer transportation costs: The study assumes that excluding costs related to fertilizer transportation to the field does not significantly impact the overall economic analysis. This assumption relies on the efficiency and reliability of the agro-dealer’s delivery system during the period of fertilizer application.

### 2.4 Economic sensitivity analysis

In the sensitivity analysis, changes in specific variables were examined to assess their impact on the outcome of a given scenario. The dataset provided included sowing dates and P levels as independent variables, gross benefit and benefit cost ratio with fertilizer application corrected against absolute control. The sensitivity analysis method typically involved focusing on altering one variable at a time while holding others constant to observe the resultant impact on the outcome. To conduct the sensitivity analysis, each variable (gross benefit and benefit to cost ratio), were isolated and their values systematically adjusted up and down by a chosen percentage of ±5%. This involved an increase by multiplying the values by 1.05 and a decrease by multiplying the values by 0.95. A sensitivity analysis is imperative as it assesses the impact of varying economic parameters. This would enhance the credibility of the economic conclusions drawn from the study.

### 2.5 Statistical data analysis

A two-way analysis of variance was carried out, incorporating economic values with sowing dates and varying P levels as the main factors. Replicates were considered as random factors in the statistical model. The representation of the factor effect model is elucidated in Equation 3. This analytical approach allows for a thorough examination of the interactions between sowing dates, P levels, and their combined influence on economic values in the study.


(3) $${\text{Y}}_{ij}={\text{μ+β}}_{i}+{\text{α}}_{j}+{\left(\text{βα}\right)}_{ij}+{\text{ϵ}}_{ij}$$

Where *Y_ij_* is the observed response variable in the *ij^th^* factor; *µ* is the overall (grand) mean; *β_i_* and *α_j_* are the main effects of the factors sowing dates and P levels, respectively, *(βα)_ij_*, and *ϵ_ij_* is the random error associated with the observation of response variable in the *ij^th^* factors.

Furthermore, a linear regression analysis was performed to explore the relationship between P levels (serving as the explanatory or independent variable) and both gross benefit and the benefit-to-cost ratio (functioning as dependent variables). This analysis aimed to predict the economic trends associated with the utilization of P-containing fertilizers in the context of cotton production. The Shapiro-Wilk test was conducted to evaluate the normality of the residuals, determining whether they followed a normal distribution. Additionally, Bartlett’s test was employed to examine the homogeneity of variances, specifically assessing whether the variances were consistent across groups for the insignificant mean differences observed in relation to the main effects of sowing dates and P levels.

## 3 Results

### 3.1 Effects of sowing dates and phosphorus on statistical parameters of gross benefit, marginal return, and benefit/cost ratio

The main effects of sowing dates were significant (*p<*0.001) on gross benefit, benefit/cost ratio, and marginal return of cotton production (**Table 2**). In addition, the main effects of P levels were significant (*p* =0.017) only on benefit/cost ratio, but not on gross benefit (*p* =0.56) and marginal return (*p* =0.783). On the other hand, the interactions between sowing dates and P levels did not have a significant effect on gross benefit, benefit/cost ratio, and marginal return (**Table 2**). Besides the insignificant main effects recorded for P levels on gross befits and marginal return, results indicated that the residuals were normally distributed and the variances were homogenous (**Table 3**).

**Table 2:** Analysis of variance for the gross benefit, benefit/cost ratio and marginal return for the data collected from economic performance of cotton at different sowing dates and phosphorus levels.

		Gross benefit			Benefit/Cost ratio					Marginal Return		
Source of variation	d.f.	m.s.	v.r.	F pr.	m.s.	v.r.	F pr.	Source of variation	d.f.	m.s.	v.r.	F pr.
Replication	2	2.84E+12	1.93		25.15	2.03		Replication	2	2.15E+12	1.68	
Dates	2	2.34E+13	15.9	<0.001	166.93	13.49	<0.001	Dates	2	2.68E+13	20.94	<0.001
Phosphorus	2	8.86E+11	0.6	0.56	65.73	5.31	0.017	Phosphorus	3	4.59E+11	0.36	0.783
Dates × Phosphorus	4	8.37E+11	0.57	0.689	2	0.16	0.955	Dates × Phosphorus	6	7.29E+11	0.57	0.75
Residual	16	1.47E+12			12.38			Residual	22	1.28E+12		
Total	26							Total	35			

Key: d.f., degrees of freedom; m.s., mean sum of squares; v.r., variance; F pr., test-F probability.

**Table 3:** Tests of normality of residuals and homogeneity of variances for the effect of phosphorus levels on gross benefit and marginal return.

Shapiro-Wilk test for Normality of Residuals		Bartlett’s test for homogeneity of variances	
*Gross benefit*			
*Test statistic W:*	0.988	*Chi-square:*	0.41
*Probability:*	0.984	*Probability:*	0.814
		*d.f.*	2
***Status:***	Normally distributed (*p >*0.05)		Homogenous (*p >*0.05)
*Marginal return*			
*Test statistic W:*	0.992	*Chi-square:*	2.31
*Probability:*	0.996	*Probability:*	0.511
		*d.f.*	3
***Status:***	Normally distributed (*p >*0.05)		Homogenous (*p >*0.05)

### 3.2 Effects of sowing dates and phosphorus on means of gross benefit, marginal return, and benefit/cost ratio

The results of the economic benefits derived from the use of P-containing fertilizer on cotton production at different sowing dates are presented in **Table 4**. With the sowing dates, the highest gross benefits were obtained during the early (Tshs. 6,679,527 equivalent to USD 2,666) and middle (Tshs. 6,861,283 equivalent to USD 2,738) sowing dates. In contrast, the lowest gross benefit (Tshs. 3,980,453 equivalent to USD 1,588) was obtained in cotton sown late. Similar to the gross benefits, the marginal return and benefit/cost ratio were in the decreasing trend of middle > early > late sowing.

**Table 4:** Means of gross benefit, benefit/cost ratio and marginal return as affected by the sowing dates and phosphorus levels.

Factors	Treatments	*GB with fertilizer	MR	BCR
		Tshs		
**Sowing dates**	25^th^ November 2022	6,679,527^a^	6,217,999^a^	18.4^a^
	15^th^ December 2022	6,861,283^a^	6,395,211^a^	18.8^a^
	4^th^ January 2023	3,980,453^b^	3,723,400^b^	11.2^b^
	*P-value*	<0.001	<0.001	<0.001
	*L.S.D. _(0.05)_*	1,212,896	957,666	3.5
**Phosphorus levels**	0	*N/A*	5,370,885^a^	*N/A*
	20	5,752,572^a^	5,452,572^a^	19.2^a^
	40	5,579,904^a^	5,209,904^a^	15.1^b^
	60	6,188,786^a^	5,748,786^a^	14.1^b^
	*P-value*	0.56	0.783	0.017
	*L.S.D. _(0.05)_*	1,212,896	1,105,817	3.5

Currency exchange rate on 13^th^ September 2023: USD 1 = Tshs. 2505.9. Key: D, date; P, phosphorus; GB, gross benefit; MR, marginal return; BCR, benefit/cost ratio; N/A, not applicable. *Means along the same column within a specific category and bearing different letter(s) differ significantly at 5% error rate.

The effects of P levels on gross benefit were not significant. A similar trend was followed for the marginal return for P applied at rates of 60 kg P ha^-1^ and 20 kg P ha^-1^, and the absolute control where no fertilizer was applied. Application of 40 kg P ha^-1^ resulted in the marginal return of Tshs. 160,981 (equivalent to USD 64) less than the marginal return obtained in absolute control. This finding suggests that the application of 40 kg P ha^-1^ results in an economic loss relative to growing cotton without any application of P-containing fertilizer. The results also showed that an application of 20 kg P ha^-1^ resulted in a significantly higher (19.2) benefit/cost ratio compared with the benefit/cost ratios recorded when P was applied at rates of 40 kg P ha^-1^ and 60 kg P ha^-1^. This finding suggests that the application of 20 kg P ha^-1^ results in higher economic benefits relative to the application of 40 kg P ha^-1^ and 60 kg P ha^-1^ in cotton. Overall, this finding provides information about the effects of sowing dates and P on the measured variables. Based on this information, sowing dates have a significant impact on all three variables, while P levels only have significant effect on marginal return.

**Figure 1:**
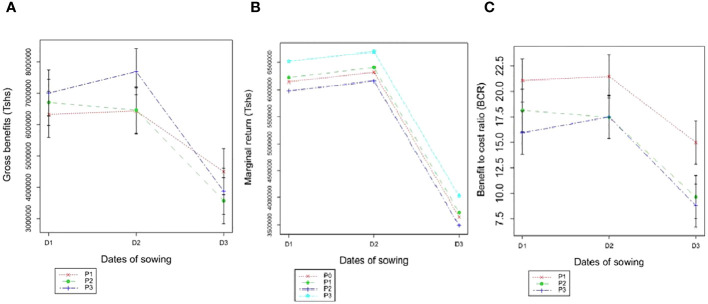
Means of gross benefit **(A)**, marginal return **(B)** and benefit/cost ratio **(C)** of cotton as affected by the interactions between sowing dates and phosphorus levels.

The interactions between sowing dates and P levels showed an insignificant effect on the trends of gross benefit, marginal return, and benefit/cost ratio (**Figure 1**). The insignificant interactions between these factors provide an exciting result that P-containing fertilizers may be applied to cotton at any sowing date and the effect is determined by the specific sowing date. This can be stretched that the economics of using P-containing fertilizers is independent of the sowing date of cotton. In assessing these economically measured variables, the benefit/cost ratio indicated that an application of P at a rate of 20 kg P ha^-1^ during middle sowing (D2) is profitable relative to early and/or late sowing.

### 3.3 Regression analysis of gross benefit and benefit/cost ratio

**Figure 2:**
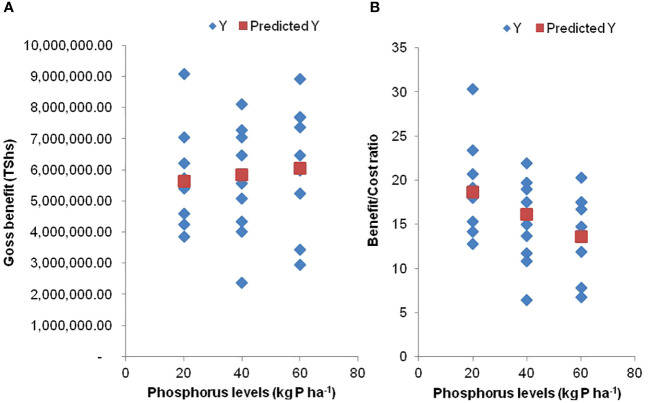
Gross benefit **(A)** and benefit/cost ratio **(B)** at varying P levels.

The results in **Table 5** show that the gross benefit derived from cotton cultivated per hectare without P application is Tshs. 5,404,207 (equivalent to USD 2,157), but the application of 1 kg P ha^-1^ is likely to increase the gross benefit by Tshs. 10,905 (equivalent to USD 4.3) but the increase is insignificant (*p* =0.61). This trend is also shown in **Figure 2A**, displaying that an increase in the amount of P applied through 40 kg P ha^-1^ and 60 kg P ha^-1^ beyond 20 kg P ha^-1^ resulted in a further increase in the gross benefit. In contrast, the results of the benefit/cost ratio show that cotton cultivation with an application of 20 kg P ha^-1^ resulted in a significant (*p<*0.001) increase in the benefit of 21.21% over the costs incurred in cotton production (**Table 6**). The results of the benefit/cost ratio also showed that a further increase in the amount of P by 1 kg P ha^-1^ applied on cotton beyond 20 kg P ha^-1^ is likely to result in a loss of 0.13 of what would be expected with low P input (**Table 5**; **Figure 2B**). These findings provide an insight that the benefit/cost ratio is more appropriate in assessing the economics of fertilizer input to cotton production instead of relying only on the gross benefit, which may be a deceiving approach.

**Table 5:** Relationship between gross benefit and phosphorus levels applied on cotton.

Regression Statistics			ANOVA					
Multiple R	0.103			*df*	*SS*	*MS*	*F*	*Significance F*
R Square	0.011		Regression	1	8.56E+11	8.56E+11	0.266	0.610
Adjusted R Square	-0.029		Residual	25	8.04E+13	3.21E+12		
Standard Error	1,793,016		Total	26	8.12E+13			
Observations	27							
	*Coefficients*	*Standard Error*	*t Stat*	*P-value*	*Lower 95%*	*Upper 95%*		
Intercept	5,404,207	912,959	5.91944196	3.54E-06	3523933	7284481		
P levels	10,905	21,131	0.51608567	0.61	-32614.5	54425.23		

Model: GB = 5,404,207 + 10,905×(P levels).

Key: df, degree of freedom; SS, sum of square; MS, mean sum of square.

**Table 6:** Relationship between benefit/cost ratio and phosphorus levels applied on cotton.

Regression Statistics			ANOVA					
Multiple R	0.403			*df*	*SS*	*MS*	*F*	*Significance F*
R Square	0.162		Regression	1	117.0	117.0	4.84	0.037
Adjusted R Square	0.129		Residual	25	604.6	24.2		
Standard Error	4.918		Total	26	721.6			
Observations	27							
	*Coefficients*	*Standard Error*	*t Stat*	*P-value*	*Lower 95%*	*Upper 95%*		
Intercept	21.21	2.5	8.47	8.15E-09	16.05	26.37		
P levels	-0.13	0.1	-2.20	0.037	-0.25	-0.01		

Model: BCR = 21.21 – 0.13×(P levels).

Key: d.f., degree of freedom; SS, sum of square; MS, mean sum of square; BCR, benefit/cost ratio.

### 3.4 Sensitivity analysis

**Figure 3:**
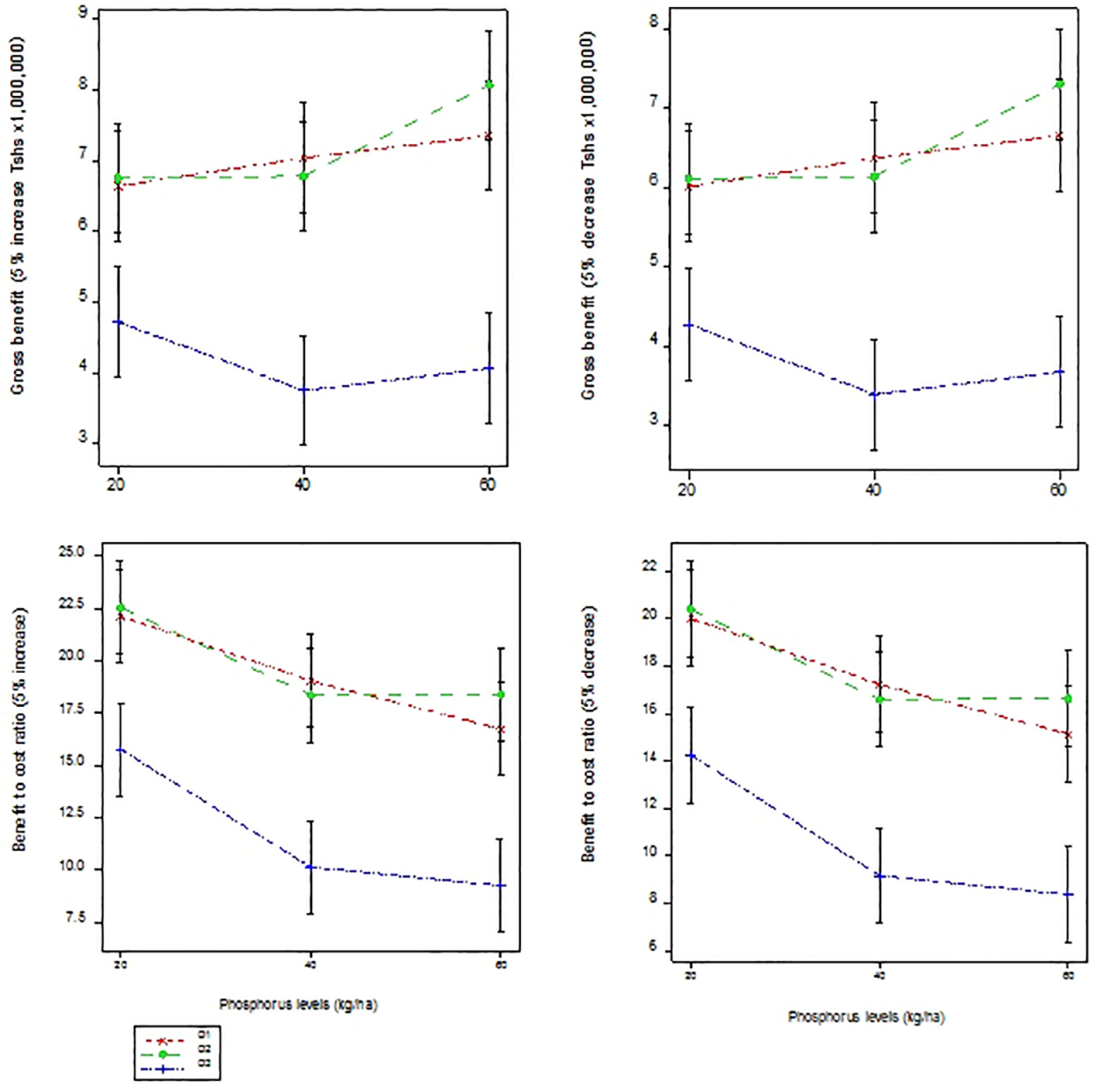
Economic sensitivity analysis of gross benefit and benefit to cost ratio as affected by sowing dates and phosphorus rates.

The results of the economic sensitivity analysis of gross benefit and benefit to cost ratio following main effects of sowing dates and P application rates are presented in **Table 7**, while interaction effects of the two factors on the same measured variables are presented in **Figure 3**.

**Table 7:** Economic sensitivity analysis of gross benefit and benefit to cost ratio as affected by sowing dates and phosphorus rates.

Factors	Gross benefit (Tshs)		Benefit to cost ratio	
	(5% increase)	(5% decrease)	(5% increase)	(5% decrease)
Sowing dates				
25^th^ November 2022	7013503^ab^	6345550^ab^	19.3^a^	17.5^a^
15^th^ December 2022	7204347^a^	6518218^a^	19.8^a^	17.9^a^
4^th^ January 2023	4179475^b^	3781430^b^	11.7^a^	10.6^a^
*LSD(0.05)*	2301741	2082528	6.5	5.9
*P-value*	0.037	0.037	0.044	0.044
*cv (%)*	16.6	16.6	16.8	16.8
Phosphorus (kg ha^-1^)				
20	6040201^a^	5464943^a^	20.1^a^	18.2^a^
40	5858899^a^	5300909^a^	15.8^b^	14.3^b^
60	6498225^a^	5879347^a^	14.8^b^	13.4^b^
*LSD(0.05)*	1093964	989777	3.3	3.0
*P-value*	0.447	0.447	0.009	0.009
*cv (%)*	0.8	0.8	2.0	2.0

Key: LSD, least significant differences of means; P-value, probability value; cv, coefficient of variation; *Means along the same column and in a specific category of factors (sowing dates and/or P rates) bearing different letter(s) differ significantly at 5% threshold based on the LSD. The GB (5% increase) Shapiro-Wilk test for Normality (Test statistic W = 0.9710; probability = 0.628) and Bartlett’s test for homogeneity of variances (Chi-square = 0.41 on 2 degrees of freedom; probability = 0.814).

The significance of the data variations was assessed by considering the least significant difference (LSD) values and *p*-values provided in **Table 7**. For gross benefit, the LSD values for sowing dates and P application rates range from approximately 1.1 to 2.3 million Tanzanian Shillings (Tshs). For the benefit-to-cost ratio, the LSD values range from about 3.0 to 6.5. For both gross benefit and benefit-to-cost ratio, the *p*-values associated with the main effects of sowing dates and P rates are 0.037 or lower. This indicates that there are significant differences between the groups. Additionally, the coefficient of variation (cv %) provides a measure of the variability relative to the mean. In this case, the cv % values for gross benefit range from 16.6% to 16.8%, and for the benefit-to-cost ratio, they range from 16.8% to 16.8%.

## 4 Discussion

### 4.1 Influence of phosphorus and sowing dates on economics of cotton

The study found that sowing dates had a significant impact on gross benefit, benefit/cost ratio, and marginal return in cotton production. There is a strong statistical relationship between sowing dates and these economic measures. In contrast, P levels were found to have a significant effect only on the benefit/cost ratio. Based on the findings of the study there is evidence to suggest that P levels influence the benefit/cost ratio, but not as strongly as sowing dates. In examining whether there were interactions between sowing dates and P levels, it was found that the interactions did not have a significant effect on gross benefit, net benefit and benefit/cost ratio. This suggests that the combined influence of sowing dates and P levels did not lead to any statistically significant differences beyond what could be explained by their individual effects. The study also found that the residuals (differences between observed and predicted values) of the main effect of P levels on gross benefit and benefit/cost ratio were normally distributed, and the variances were homogenous. This finding indicates that the statistical analysis conducted in the study for P levels met certain assumptions, such as the normality of residuals and homoscedasticity (equal variances), which are important for the reliability of statistical tests (Williams et al., 2019; Lee, 2020).

This study reports the results of an agricultural study, highlighting the significant impact of sowing dates on various economic measures related to cotton production, the influence of P levels primarily on the benefit/cost ratio, and the lack of significant interaction effects between sowing dates and P levels. However, Shapiro-Wilk and Bartlett’s tests performed in this study for P levels represent the fulfilment of important statistical assumptions, reinforcing the validity of the study’s findings. These findings can be used to make informed decisions about cotton production practices and resource allocation.

The results highlight the importance of sowing date in maximizing cotton yield and economic returns. Early and middle sowing, represented by D1 and D2, consistently outperform late sowing (D3) across gross benefit, net profit and benefit/cost ratio. While P levels show differences in benefit/cost ratio, there is no clear statistical significance in yield or the unexplained metric among the levels (Sawan, 2018; Mudassir et al., 2022). This may suggest that the economic benefits associated with different P levels are not solely driven by yield but by other factors not explicitly measured here (van de Wiel et al., 2016; Mai et al., 2018). Studies conducted by other researchers demonstrate how the market prices of cotton impact planting decisions on optimal planting dates to maximize profits (Singh et al., 2017; Boyer et al., 2020; Sankaranarayanan et al., 2020). Sankaranarayanan et al. (2020) found that the sowing date of cotton was significantly influenced by the total variable costs, gross benefit, and net return. According to Sankaranarayanan et al. (2020), early and middle sowing of cotton were similar and significantly higher in terms of total variable costs, gross benefit, and net return compared with the late sowing based on the onset of the rainy season. Zhang et al. (2017) found that the sowing date influenced the yields and economics of cotton, with early sowing during the onset of the rainy season increasing N, P, and K absorption by the plant, seed cotton, and lint yield of up to 6.5 t ha^-1^. According to Zhang et al. (2017), late sowing of cotton resulted in poor lint production, low economic index, and other yield and economic attributes.

Fei et al. (2022) found a strong and positive relationship between cotton economic growth and human capital in the smallholder farming systems of Benin. Ahmad Anjum et al. (2015) found the benefit-cost ratio, net benefit, and marginal rate of return to be differently influenced by cotton varieties and planting density when sown early during the onset of the rainy season. Based on the existing literature of similar studies conducted elsewhere, and the results of the present study the optimum sowing date for cotton in the study area is during the third week (towards the end of November) from the onset of the rainy season. The results of the present study also provide valuable insights for farmers and researchers, suggesting that optimizing sowing dates can have a significant impact on crop production and profitability. In practice, these findings suggest that farmers may also benefit from early sowing, but careful consideration should also be given to P levels, especially with regard to economic returns. Further research could explore the interaction between sowing dates and various P levels to provide more comprehensive recommendations for cotton production in the study area.

The results of the regression analysis show that applying P in cotton production in the study area is expected to have a general increase in gross benefit but this increase is deemed statistically insignificant. This suggests that while there is a numerical increase in gross benefit with P application, it is not strong enough to be considered statistically meaningful. A graphical representation of the relationship between the amount of P applied (ranging from 20 kg to 60 kg P ha^-1^) and gross benefit shows that increasing P beyond 20 kg P ha^-1^ results in further increases in gross benefit. However, these increases are not statistically significant implying that while there might be a trend it is not strong enough to conclude that increasing P significantly boosts gross benefit of cotton. Shifting the focus to the benefit/cost ratio, applying 20 kg P ha^-1^ leads to a significant increase in the benefit/cost ratio, which is a 21.21% improvement over the costs incurred in cotton production. This finding suggests that investing in 20 kg P ha^-1^ yields a favourable return on investment, and it is statistically significant. The study also highlights that increasing the amount of P beyond 20 kg P ha^-1^ does not lead to further improvements in the benefit/cost ratio. This finding suggests that there is a diminishing return, as a further increase in P by 1 kg P ha^-1^ results in a loss compared to what would be expected with lower P input. This finding also underscores the importance of not overapplying P in cotton, as it can lead to economic inefficiency (Masud et al., 1985; Iqbal et al., 2020; Romero-Perdomo et al., 2021). This finding emphasizes the importance of using the benefit/cost ratio as an indicator for assessing the economic viability of fertilizer input in cotton production. It also suggests that relying solely on gross benefit can be misleading, as it does not account for the cost side of the equation. The benefit/cost ratio provides a more comprehensive picture of the economic efficiency of P application in cotton production (Ali and Ahmad, 2021; El Wali et al., 2021). Ali and Ahmad (2021) reported that the economics of cotton production is well realized through the benefit/cost ratio. According to Ali and Ahmad (2021), the benefits derived from investing in inputs depend on how easily the inputs are implemented at a reasonable cost. Low P inputs in cotton production with a higher benefit/cost ratio have implications of reduced environmental impact of synthetic fertilizers (Bindraban et al., 2020). According to Constable and Bange (2015), cotton plants can utilize 3 to 4 kg of P per bale of lint (or 30 to 40 kg P ha^-1^), translating into about 5 t ha^-1^ of cotton. Nachimuthu et al. (2022) reported that P has been applied in commercial cotton fields at rates of 20 to 40 kg P ha^-1^ since 2000.

The study underscores the significance of using the benefit-to-cost ratio as a comprehensive indicator for assessing the economic viability of fertilizer input in cotton production. It challenges the reliance solely on gross benefit, highlighting its potential for being misleading as it overlooks the cost aspect of the equation. The benefit-to-cost ratio, as advocated by previous research, offers a more nuanced understanding of the economic efficiency of P application in cotton production. Furthermore, the economic realization of cotton production, as noted by Ali and Ahmad (2021), hinges on the ease of input implementation at reasonable costs. Lower P inputs with a higher benefit-to-cost ratio may not only be economically efficient but also have implications for reducing the environmental impact of synthetic fertilizers (Pradel and Aissani, 2019; Garske and Ekardt, 2021). Considering the recommended P utilization by cotton plants and historical application rates, the study contributes to the ongoing discourse on optimizing P application in cotton production. However, a potential research gap emerges in the absence of an exploration into the specific factors influencing the statistical insignificance in gross benefit increase with P and a more in-depth analysis of the diminishing returns beyond 20 kg P ha^-1^ in the benefit-to-cost ratio. Addressing these aspects could enhance the depth and completeness of the study’s findings.

The economic sensitivity of utilizing P at different sowing dates for cotton production followed similar trend to ordinary analysis. Sowing dates are crucial determinants influencing the growth and yield of cotton crops, with the generated information exhibiting variations in gross benefit and benefit-to-cost ratio to be associated with altering sowing dates and P application levels. Focusing on sowing dates, the results unveil distinct economic outcomes. Cotton sown on the 25^th^ November 2022 manifests the highest gross benefit, followed closely by that sown on the 15^th^ December 2022. Conversely, cotton sown on the 4^th^ January 2023 yielded significantly lower gross benefits. This discrepancy shows the critical importance of timely sowing in maximizing economic returns in cotton cultivation (Fei et al., 2022). Furthermore, examining the benefit-to-cost ratio across sowing dates reveals a similar trend. Cotton sown earlier demonstrates superior benefit-to-cost ratios compared to those sown later. This finding shows the advantage of early sowing in optimizing the economic efficiency of cotton production, likely due to extended growing periods and better utilization of resources (Qi et al., 2023).

In addition to sowing dates, the application of P plays a vital role in determining economic outcomes in cotton farming. The findings of the present study show varying gross benefits and benefit-to-cost ratios corresponding to different levels of P application. Generally, higher levels of P application result in increased gross benefits and improved benefit-to-cost ratios, emphasizing the economic significance of P supplementation in cotton cultivation (Romero-Perdomo et al., 2021).

### 4.2 Environmental impacts associated with phosphorus application

P and N are indispensable for fostering plant growth, including in cotton plants (Romero-Perdomo et al., 2021; Khan et al., 2023). However, their extensive application in agriculture, particularly in cotton farming, raises profound environmental concerns, encompassing water pollution and soil degradation (Jwaideh et al., 2022). The application of P, typically in the form of diammonium phosphate, can engender numerous environmental challenges. Excessive P usage, notably at higher rates such as those observed in the present study (40 and 60 kg P ha^-1^), can trigger runoff and leaching, leading to P enrichment in adjacent water bodies (Romero-Perdomo et al., 2021; Khan et al., 2023). This influx of P-rich runoff into rivers, lakes, and streams can incite eutrophication, prompting algal overgrowth. Consequently, oxygen depletion transpires, culminating in fish mortality and disruption of aquatic ecosystems (Jwaideh et al., 2022).

Furthermore, P runoff from cotton fields contributes to freshwater quality degradation, jeopardizing drinking water sources and recreational areas (Romero-Perdomo et al., 2021). The accumulation of P in water bodies fosters the proliferation of harmful algal species, which release toxins detrimental to aquatic life and human health (Khan et al., 2023). In tandem with P, N application, often in the form of urea, presents environmental hurdles in cotton cultivation. Unrestrained nitrogen application, lacking adequate management practices, can lead to nitrate leaching into groundwater and surface water bodies (Anas et al., 2020). Nitrate contamination in drinking water sources poses grave health hazards, especially to vulnerable demographics such as infants and pregnant women, potentially inducing methemoglobinemia or “blue baby syndrome” (Manassaram et al., 2006; Temkin et al., 2019; Grout et al., 2023).

Furthermore, nitrogen fertilizer application contributes to atmospheric pollution through nitrous oxide (N_2_O) emissions, exacerbating climate change and ozone depletion (Thomson et al., 2012; Aryal et al., 2022; Schomberg et al., 2023). Soil degradation emerges as another pressing issue linked to excessive P and N application in cotton farming (Honfoga, 2018; Awadelkareem et al., 2023). Prolonged imbalanced nutrient application can precipitate soil acidification, nutrient imbalances, and diminished soil fertility, imperilling the long-term viability and sustainability of cotton farming systems (Uwiragiye et al., 2023; Salakinkop et al., 2024).

To mitigate these environmental ramifications, embracing sustainable agricultural practices is imperative. Precision nutrient management, cover cropping, and buffer strips can curtail nutrient runoff while fortifying soil health in cotton production systems (Nuruzzaman Manik et al., 2019; Wang et al., 2023). Furthermore, promoting integrated nutrient management strategies can optimize nutrient utilization efficiency while mitigating the environmental risks posed by P and N application in cotton farming (Paramesh et al., 2023; Wang et al., 2023; Kumar et al., 2024). The approaches addressed in the present study align with various Sustainable Development Goals (SDGs), notably SDG 2 (Zero Hunger), SDG 6 (Clean Water and Sanitation), SDG 12 (Responsible Consumption and Production), and SDG 15 (Life on Land). In attempts to address the environmental impacts of P and N application in cotton cultivation, these practices contribute to broader sustainability objectives, fostering resilient agricultural systems while safeguarding natural resources for future generations.

## 5 Conclusions and recommendations

The study aimed to assess the economic implications of different sowing dates and P application levels on cotton production in Msilale village, Tanzania. The results indicated that early sowing dates, particularly on the 25^th^ of November and 15^th^ of December resulted in significantly higher cotton yields compared to later sowing dates. Additionally, application of 20 kg P ha^-1^ showed the highest net profits and benefit-to-cost ratios, with no significant improvements observed with higher P levels. Based on these findings, farmers in Msilale village and similar regions should prioritize early sowing dates, ranging from late November to mid December, to maximize yields and economic returns. Moreover, adopting 20 kg P ha^-1^ can ensure cost-effectiveness and optimal economic outcomes. Overapplication of P beyond this level does not yield substantial benefits, emphasizing the need for careful management of fertilizer inputs to avoid unnecessary expenses.

Based on the findings of the present study, it is recommended that farmers in the study area integrate these findings into their cotton production practices. Agricultural extension services and governmental agencies can play a crucial role in disseminating this information and providing support for implementation. Furthermore, ongoing research and monitoring are essential to continually refine and adapt recommendations based on evolving agricultural conditions and market dynamics.

## Data availability statement

The original contributions presented in the study are included in the article/supplementary material. Further inquiries can be directed to the corresponding author.

## Author contributions

JT: Conceptualization, Data curation, Formal analysis, Investigation, Methodology, Project administration, Resources, Software, Validation, Visualization, Writing – original draft, Writing – review & editing. EN: Supervision, Validation, Writing – review & editing. GT: Supervision, Validation, Writing – review & editing.
